# Magnetic resonance imaging of Joubert syndrome associated with Dandy-Walker malformation: pathognomonic imaging

**DOI:** 10.11604/pamj.2022.42.55.31984

**Published:** 2022-05-19

**Authors:** Jihane Habi, Mohamed Mahi

**Affiliations:** 1 Department of Radiology, Faculty of Medicine, Mohammed VI University of Health Sciences, Cheikh Khalifa International University Hospital, Casablanca, Morocco

**Keywords:** Joubert syndrome, Dandy-Walker, imaging

## Image in medicine

An eight-month-old infant was referred to our institution to investigate psychomotor retardation. The clinical finding found ataxia. A magnetic resonance imaging (MRI) was indicated and performed. Sequence T1 in the axial and coronal plane (A,B) revealed a long thick superior cerebellar peduncle (yellow arrow) and an interpeduncular fossa (white arrow), reminding the molar tooth sign (MTS). Furthermore, sequence T1 in the sagittal plane (C) showed hypoplasia of the vermis (red arrow) and global ascension of the tentorium cerebelli (red star). Finally, the diagnostic of Joubert syndrome associated with Dandy-Walker malformation was confirmed by pathognomonic imaging. The family patient was informed and received the best medical assistance. Joubert syndrome is a rare recessive ciliopathy, diagnosed by a triad in cross-sectional imaging (scanner/MRI): thick and straight superior cerebellar peduncles, deep interpeduncular fossa, and hypoplastic/dysplastic vermis forming the “molar tooth sign”. Dandy-Walker syndrome is defined as rare malformation, presented in MRI as upward displacement of the tentorium, torcular and lateral sinuses and anterior-posterior enlargement of the posterior fossa. In effect, the association between the Joubert syndrome and Dandy-Walker malformation is even rarer. Joubert syndrome is often combined with liver, renal and eye disease. Its treatment is cumbersome as well as multidisciplinary depending on the organs affected, hence imaging is important to the precise diagnosis, thereby reduction its medical complications.

**Figure 1:**
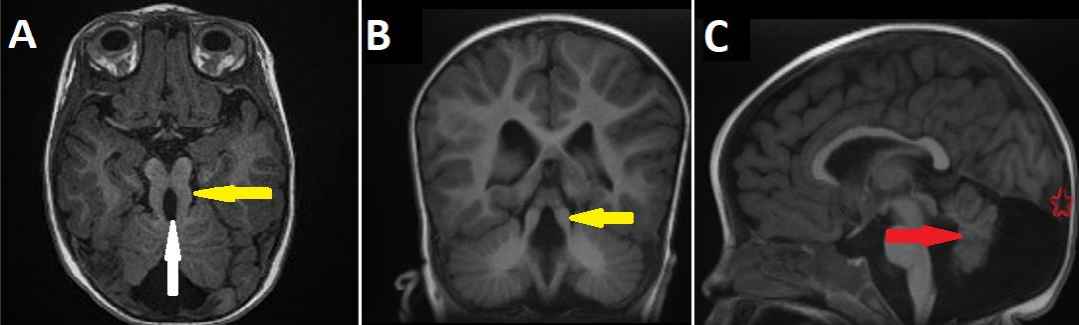
MRI of Joubert syndrome associated with Dandy-Walker malformation; axial plane (A), coronal plane (B), and sagittal plane (C)

